# The importance of children and young person involvement in scoping the need for a paediatric glucocorticoid-associated patient reported outcome measure

**DOI:** 10.1186/s41927-022-00312-9

**Published:** 2022-10-15

**Authors:** S. Singhal, E. M. D. Smith, L. Roper, C. E. Pain

**Affiliations:** 1 Institute in the Park, Alder Hey Children’s Hospital, Institute of Life Course and Medical Sciences, University of Liverpool, Eaton Rd, Liverpool, L12 2AP UK; 2 Department of Paediatric Rheumatology, Alder Hey Children’s NHS Foundation Trust, Liverpool, UK; 3 Department of Clinical Health Psychology, Liverpool University Hospital Foundation Trust, Liverpool, UK

**Keywords:** Patient and public involvement, Patient reported outcome, Glucocorticoid, Children and young people

## Abstract

**Background:**

For many children and young people (CYP) with paediatric rheumatic conditions, glucocorticoid medications and their associated side-effects have a substantial impact on disease experience. Whilst there are physician-rated measures of glucocorticoid toxicity, no parallel patient reported measure has been developed to date for CYP with rheumatic disease. This manuscript describes a series of public patient involvement (PPI) events to inform the development of a future paediatric glucocorticoid-associated patient reported outcome measure (PROM).

**Methods:**

One large group PPI event was advertised to CYP with experience of glucocorticoid medication use and their parents through clinicians, charities and existing PPI groups. This featured education on the team’s research into glucocorticoid medication and interactive polls/structured discussion to help participants share their experiences. Further engagement was sought for PPI group work to co-develop future glucocorticoid studies, including development of a glucocorticoid associated PROM. Quantitative and qualitative feedback was collected from online questionnaires. The initiative was held virtually due to the Covid-19 pandemic.

**Results:**

Nine families (n = 15) including 6 CYP joined the large group PPI event. Online pre-attendance and post-attendance questionnaires showed improvement in mean self-reported confidence [1 = not at all confident, 5 = very confident] in the following: what steroid medications are (pre = 3.9, post = 4.8), steroid side effects (pre = 3.8, post = 4.6), patient-reported outcome measures (pre = 2.0, post = 4.5), available research on steroids (pre = 2.2, post = 3.5). Five families (n = 7) were involved in a monthly PPI group who worked alongside the research team to identify priorities in glucocorticoid research, produce age-appropriate study materials, identify barriers to study participation (e.g. accessibility & convenience) and recommend appropriate modalities for dissemination. The participants found discussing shared experiences and learning about research to be the most enjoyable aspects of the initiative.

**Conclusions:**

This PPI initiative provided a valuable forum for families, including young children, to share their perspectives. Here, the authors explore the effective use of PPI in a virtual setting and provide a unique case study for the involvement of CYP in PROM development. The monthly PPI group also identified a need for the development of a new PROM related to glucocorticoid medication use and provided unique insights into how such a study could be structured.

**Supplementary Information:**

The online version contains supplementary material available at 10.1186/s41927-022-00312-9.

## Background

Patient and public involvement (PPI) allows families to use their own experience to contribute meaningfully to health research and learn about research processes and findings. Involvement of patients and public follows the key principle of research being carried out ‘with’ or ‘by’ members of the public rather than ‘to’, ‘about’ or ‘for’ them [1]. PPI in research has been described as a moral imperative as enshrined in the UN Convention of the Rights of the Child [2, 3] and evidence reports PPI may improve recruitment and retention within clinical trials [4].

PPI in healthcare research has increasingly become a requirement by regulatory and funding bodies [5]. UK National standards on PPI involvement in research foster inclusivity of patients, carers and the wider community in health and social care research [6]. Similar standards exist in other countries, such as the US Food and Drug Administration who mandate PPI in the development process of PROMs [7]. Despite this, a growing need for high-quality, meaningful PPI involvement in all forms of research has been identified, including in PROM development, compared to tokenistic or ‘tick box’ PPI [2, 8, 9].

Patient reported outcomes prioritise the outcomes most relevant to patients and thus cannot be developed without a central focus on the patient perspective. PROMs typically take the form of a questionnaire or series of questions that are completed by patients to give an insight into a patient’s overall functional status or wellbeing [10]. They were initially developed for use in clinical research but over time their use in a routine healthcare setting has become commonplace [11]. PROMs have been used to improve the quality of healthcare in a number of ways: assisting clinicians to provide better and more patient centred care; assessing and comparing the quality of providers; and providing data for evaluating practices and policies [12]. Since their development, PROMs have continued to have an increasing scope in healthcare, reflecting a growing recognition of patient health-related quality of life (HRQOL) as a valuable outcome [13].

An important component of HRQOL in CYP with rheumatic diseases is the use of glucocorticoid medications and its associated side effects. Glucocorticoid medications offer potential for rapid clinical improvement, but frequently lead to side effects that adversely impact HRQOL. Many studies looking at the patient experience within paediatric rheumatic diseases, have found the burden of glucocorticoid treatment to be a recurring theme [14–17]. These range from short-term effects that may go unrecognised by clinicians (e.g., weight gain, anxiety, skin changes and poor sleep) to long-lasting adverse events (e.g., delayed growth and puberty, diabetes, loss of bone mass and fractures) [15]. Despite widespread recognition of the importance of limiting glucocorticoid use, there are no currently available patient reported outcome measures (PROMs) that can be used to capture the patient experience of glucocorticoid treatment in paediatric rheumatic disease.

The research team hosted a series of PPI events focused upon the use of glucocorticoid medication in children and young people (CYP), to share experiences of glucocorticoid treatment, learn about the research team’s work related to glucocorticoids and create a framework for the future development of a paediatric glucocorticoid-associated PROM. This paper also describes the methodological barriers and facilitators to PPI in the virtual setting, including the co-development of each aspect of the research process. Finally, the authors provide a model for PPI in development of PROMs.

## Methods

### Large group PPI event

The initial large group PPI event was advertised to parents of CYP with experience of glucocorticoid medication use, through clinicians (n = 4), charities (n = 9) and patient groups (n = 2), including their associated social media channels. From these, nine families from across England and Wales with CYP who had required glucocorticoid treatment volunteered to participate in the event: 3 with Nephrotic Syndrome, 2 with Systemic Lupus Erythematous (SLE), 1 with Juvenile Idiopathic Arthritis (JIA), 1 with Behçet’s Disease, 1 with Cystic Fibrosis (CF) and 1 with Allergic Bronchopulmonary Aspergillosis (ABPA). There were a total of 15 participants. The ages of the CYP ranged from 2 to 17 years with 3 adults over the age of 18 who had past experience of glucocorticoid use as CYP. Participants had used glucocorticoid medication through a variety of administration routes (oral, intravenous, intra-articular, topical, inhaled). The duration of glucocorticoid medication use varied from < 1 year to > 10 years.

The event was held virtually on Zoom due to Covid-19 restrictions. During the session, the research team shared findings from four ongoing studies involving glucocorticoid medications. Interactive polls and structured discussion were used to help participants share their experiences of glucocorticoid medication. Pre- and post-attendance online questionnaires were used to collect quantitative and qualitative feedback about the event.

### Monthly PPI group meetings

CYP and their parents from the large group PPI event were invited for participation in a series of monthly smaller PPI groups, held April-June 2021, on Saturdays so that CYP could participate. Five families committed to the full series of 3 monthly PPI events, with a total of eight participants. The ages of the CYP ranged from 7 to 17 with 1 adult with previous experience of glucocorticoid treatment as a CYP. The conditions necessitating glucocorticoid use included SLE, Behçet’s disease and Nephrotic Syndrome.

The aim of the meetings was to provide input into glucocorticoid-associated clinical studies and to inform the development of a future PROM evaluating glucocorticoid associated HRQOL in paediatric patients. Both CYP and parents were compensated for their time as per the NIHR INVOLVE guidelines [6]. The small group meetings were also held virtually on Zoom, and composed of education segments, interactive tasks and structured discussions. The group was given education on how clinical research is performed and the importance of study design through short presentations and animations [18]. Feedback from the participants was collected following each meeting using online questionnaires and small group discussion to inform future sessions.

## Results

### Large group PPI event

Of the nine families that took part in the large group event, most participants (86%) reported that they were ‘likely’ or ‘very likely’ to attend future PPI events and that they were ‘likely’ or ‘very likely’ to recommend the event to friends and family. Online pre-attendance and post-attendance questionnaires showed the education from the research team on their ongoing studies led to improvement in mean self-reported confidence in their knowledge of glucocorticoid medication and associated research [1 = not at all confident, 5 = very confident] in the following: what steroid medications are (pre = 3.9, post = 4.8), steroid side effects (pre = 3.8, post = 4.6), patient-reported outcome measures (pre = 2.0, post = 4.5), available research on steroids (pre = 2.2, post = 3.5).

**Fig. 1:**
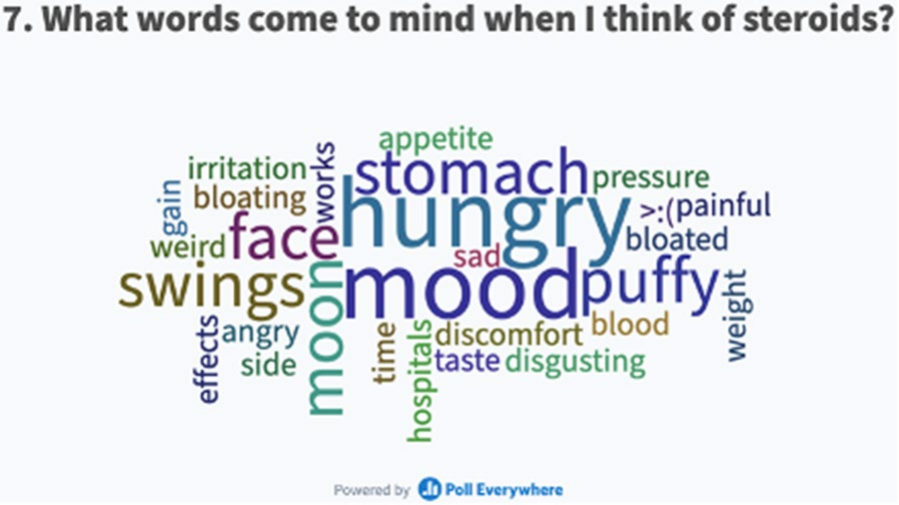
‘Word cloud’ from interactive poll at large group CYP glucocorticoid medication PPI event

The interactive polls and quizzes were used to encourage discussion and allow families to share their experiences with one another. Submissions of word associations with glucocorticoid medication formed a ‘word cloud’ which can be seen in Fig. 1. This showed a focus on glucocorticoid medication side-effects with the predominant words being ‘hungry’, ‘mood’ and ‘stomach’.

During structured small group discussion, CYP and their parents shared their diverse experiences and views in relation to glucocorticoid medication. Many reported benefits including rapid improvement in their disease, reduced pain and improved mobility. However, there were a greater number of negative effects reported, particularly those impacting upon health-related quality of life, including effects on body image, school, mood and relationships with others. This highlighted the need for a future PROM to capture both positive and negative aspects of glucocorticoid use.

### Monthly PPI group events

**Fig. 2:**
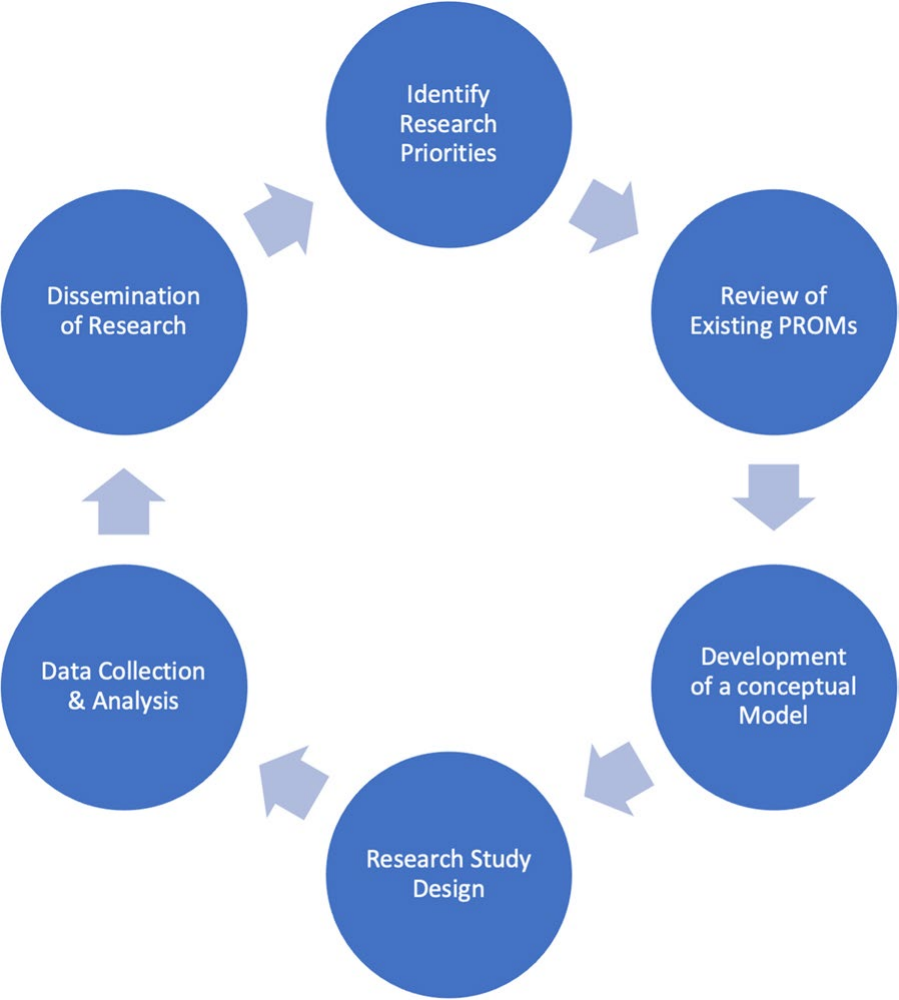
Areas identifying PPI involvement in PROM development

Over the course of three two-hour virtual meetings the group of five families were able to offer valuable insight on all aspects of the research and PROM development process through tasks and structured discussion (Fig. 2): identifying research priorities, reviewing existing PROMS, development of a conceptual model, research study design, data collection, dissemination of research. Participant IDs have not been included with quotes to maintain anonymity.

#### Identifying research priorities

The participants used their lived experience to offer insight into priorities in glucocorticoid-associated research. This was condensed into a ‘mind map’ which would be used to inform priorities for future clinical studies (Additional file 1). Key priorities identified included whether less glucocorticoid medication could be used (i.e., available alternatives and reduced duration/dosage regimes) and how information on glucocorticoid side-effects / treatment strategies could be better communicated.

#### Evaluating existing PROMs

Three PROMs which may be used to evaluate health-related quality of life in CYP who use glucocorticoid medication were evaluated: Kindl-R [19], the PedsQL Rheumatology Module [20] and the QuESt Tool [21]. The group found the Kindl-R and Peds QL Rheumatology Module measures clear and easy to use but had limited utility in detecting glucocorticoid related effects, such as change in body image and appetite. The QuEST tool was developed to measure glucocorticoid-associated QOL in children with acute lymphoblastic leukaemia. Although this featured a number of features specific to glucocorticoid use, the group felt it did not capture the benefits of glucocorticoid use in regaining mobility/QOL and rapid control of the primary disease. They identified the need for a unique PROM for measurement of glucocorticoid-associated HRQOL.

#### Development of a conceptual model

Many PROMs develop a conceptual and theoretical framework which forms domains into which items may be categorized. PPI involvement in the development of a conceptual model further ensures validity of the PROM in measuring the intended outcome [9]. The group voted on domains for inclusion, which were drawn from previously reviewed PROMs and group discussion. ‘Pain’ was excluded as a domain, as it did not reflect the experience of participants where pain was not a primary symptom e.g. nephrotic syndrome. Some of the domains that were identified for inclusion were: emotional effects (“How steroids make me feel”) and ‘social participation’ (“what I can/can’t take part in”).

#### Study design

**Fig. 3:**
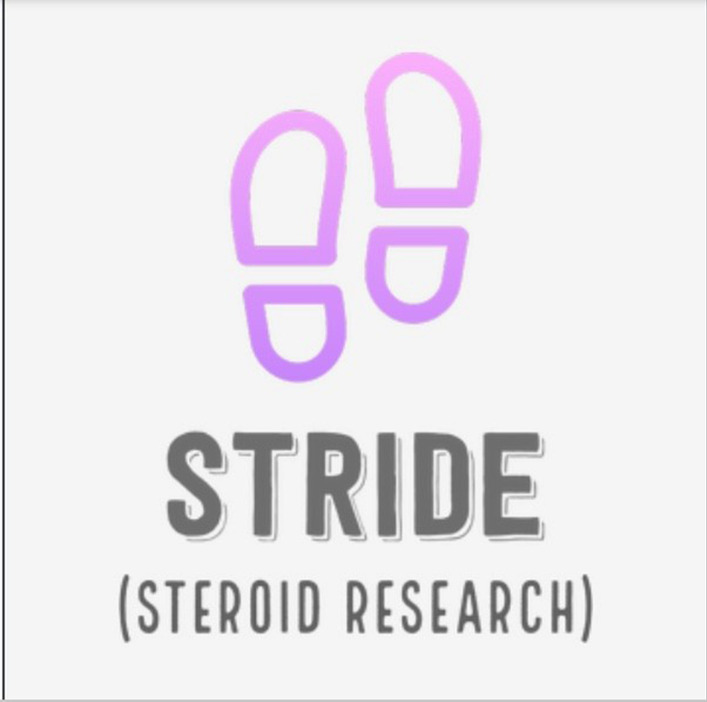
STRIDE ‘Steroid Research’ Study Logo

The PPI group generated three potential names for the PROM that were then voted on by the group. The winning name was STRIDE (STeRoID rEsearch). This was felt to represent the ‘journey’ of using glucocorticoid medication and its associated side effects. A study logo was designed showing a pink footprint (Fig. 3). The parent of a CYP who generated the logo said*: “I wanted it to be like ‘taking it in your stride with the steroids’ and [CYP] really, really liked the little pink feet.”*

The participants provided advice on how to advertise the glucocorticoid PROM development study to other CYP and their families to facilitate recruitment. They identified using social media, clinicians, and targeting existing patient groups as effective methods of recruitment. A parent said, *“Sometimes [social media] is more accessible, because, for instance, my daughter's severely visually impaired. So she can use social media to adjust the size and the contrast, whereas quite often a form in hospital won't be provided in a format she can see.”*

Important information to include within study materials was considered. This included the immediate concerns of CYP and practical concerns of parents: *“First thing my daughter wants to know is ‘will it hurt?’” “If you live a long way from a hospital ‘will you have to make special trips in?’ and ‘how often?’ is quite important to understand before you join a study”.* The group also expressed a desire for information on the route of feedback from the study. A parent said, *“It'd be nice to know that you're actually going to get the feedback from the study. Once the study’s taken place, what's actually come of the study? You've got all these results from it—what's going to happen with those results?”.*

When discussing study material, the group advised that using animations or videos were more accessible than leaflets with large blocks of text. A CYP said *“I'd be more likely to watch the video then read something”.* A parent reported *“I think too much text is always off putting, so to break it up with summaries and images is always good for me.”*

#### Data collection

Small group discussion focused on barriers to study participation in further development of the PROM, including cognitive interviewing for relevance, ease of use and readability. The group discussed the structure and setting of the proposed interviews.

There was a disparity between older CYP, who preferred a focus group setting, and younger CYP, for whom single interviews were thought to be more appropriate*: “From my perspective, having a seven-year-old, I think more one-to-one basis would probably work better with him … I think lots of younger children, especially my son, would probably copy what other little children would say rather than have his own point of view if he was in a group”.*

One CYP also highlighted the importance of siblings as part of the family unit, who are equally affected by of glucocorticoid treatment*: “I think it'd be quite interesting to have siblings’ point of view because obviously they're living with you and they’re similar ages and seeing the impact of what's happening. Because I've got three siblings that are very similar ages to me and were going through seeing me on steroids from the age of 10.”*

Flexibility was recommended to allow the greatest numbers of CYP and parents to take part. There was also a preference for a virtual setting for the interviews, perhaps in part due to the online setting of the PPI group*: “I see [CYP]’s consultants face to face and we do video conference calls. A lot of people are on social media. Especially if they can't get somewhere, it might be easier to do from home.”*

#### Dissemination of research findings

The group discussed barriers to the dissemination of research and generated ideas on how these can be overcome. Participants within the group who had previously taken part in research studies reported feeling dissatisfied with a lack of feedback on the outcomes of the research study. A parent said: *“We did sign up for [the study], because we thought it's only going to help other children, but we've never really heard anything else from it”.*

The time taken to complete research studies was identified as a barrier, as was the lack of ‘lay summaries’ of peer-reviewed research, written in language that is accessible to patients. One CYP said *“They're not very accessible. Like if I want to read about a study like and I'll try and find it in a paper and then my [family member]’s a doctor, so I’ll get my [family member] to read it, and tell me what it says”.* The group generated ideas on how research findings could be presented in a way that is meaningful to families*.* A CYP said *“I think sometimes statistics can be quite scary. Like I look at some statistics about lupus sometimes and I'm like ‘Ah, that's really scary. I don't like it’. Whereas anecdotes you can relate to them […] like having a little video with different people telling little stories.”*

A lack of age-appropriate language was also viewed as a barrier*.* A parent reported *“Children, if they've inputted might like to read the findings, but what they would want to read is probably quite different to maybe what a 25 year old would want to read. [..] Maybe a bit more creative, but a bit shorter. It depends on what the research says, you might want to not give some of the more scarier elements.”* The group identified utility in having different summaries of research findings for different ages/levels of interests.

#### Evaluation of monthly PPI group

Feedback for the monthly PPI group was collected through online questionnaires and group discussion. When evaluating the initiative, the participants reported that they enjoyed sharing their experiences with other members of the group and the research team. One CYP said *“I'm just gonna say it's been really easy to be open and honest in this group, because I just haven't felt like anyone's judging me or anything like that. So it's been really easy to open up.”* A parent said, *“It's been a lovely group. Everyone's been very open and honest. Very respectful”*. The effect of the positive relationships within the group was also noted by the research team involved who felt the project offered a valuable and unique viewpoint into glucocorticoid medication use and its side effects. One member of the research team said *“Some of the things I would never have thought of. It opens up your mind to different options going forward.”* and another said *“I've really, really changed my perspective on a lot of things”*.

The group also enjoyed learning about how research is performed. A CYP said *“I’ve enjoyed learning, it’s been quite fun”* and a parent said *“We have never done research before, so learnt a lot”*. The group however felt that the meetings would have benefitted from further diversity in the group, when asked how the initiative could have been improved*.* A parent said: *“There wasn't any men or [older] boys, though, it would be good to have maybe a bit more of a mix of people”.*

The participants reported that they would like to continue meeting regularly both for further updates on the study and to gather with the other participants and researchers. Simple feedback between patients and researchers can improve the involvement process, spur mutual learning, and change researchers’ mind-sets and future practice [22].

Both the large group PPI event and the monthly PPI group event series created a number of useful resources for use in future research related to glucocorticoid medication. This will be foundational to the creation of a PROM focused on health-related quality of life in CYP using glucocorticoid medication.

Additional file 2 includes a plain language summary of the findings from this PPI initiative.

## Discussion

Families, including CYP who take glucocorticoid medication, must be included in a meaningful way from the beginning of research studies, in order for their voice to be considered when research is designed. This is particularly so in the case of CYP, in order that their voices remain central to paediatric research. Effective PPI is characterised through the UK National Standards for Public Involvement as: Support & Learning, Impact, Communications, Inclusive Opportunities, Working Together, Governance [6]. The authors discuss how many of these features of effective PPI involvement were included within this virtual PPI initiative, whilst highlighting some of the barriers to implementation, and also outline how PPI can be used to inform future co-development of a PROM.

High quality PPI, that meets the participants where they are in terms of knowledge and understanding, has the ability to offer novel and innovative insights into the research process [9, 23]. To achieve this, participants should be supported with adequate training and tools to understand the research process [24] and therefore be able to meaningfully contribute to nuanced discussions. Education on the process of research also aims to improve public trust in the outcomes of health and social care research studies. Furthermore, this was found to be one of the most enjoyable aspects of the initiative by the participants. Another strength of the initiative was the range of expertise working alongside the PPI group, including doctors with expertise in participants’ illnesses, and a psychologist with expertise in qualitative and PPI work. Members of both the large group event and the monthly PPI group were offered support from the research team outside of the PPI setting and signposted to further resources, although this was not adopted.

Participants were compensated for their time as per the NIHR INVOLVE guidelines [25] which is the nationally recognised payment for people who are patient experts and who are giving their time to support research. It was important to the research team to compensate the children in the same way as the adults, giving children equal value in the group as co-creators of the research process. Financial cost and time investment are two of the most commonly noted barriers to extensive PPI involvement in clinical studies [2, 26]. Some have reported frustration that funding to support PPI prior to funding applications could be difficult to obtain and that local NHS and Higher Education Institution administrative practices may slow down prompt reimbursement and payment [27].

The lack of suitable ways of disseminating research findings to the wider public was identified as a particular barrier by the PPI group. Dissemination may be facilitated by patient groups and charity organisations [28], or through the use of ‘patient advocates’ [29]. Large group PPI events, as the one described here, represent another route to disseminate key research findings to relevant patient groups [30].

The virtual setting of the large group event and monthly meetings presented both opportunities for inclusive research, and also challenges. For example, a benefit was that participants could raise their hand, use the chat box, or shout out when they wanted to contribute. This was then noticed by the facilitator (a medical doctor or psychologist) so CYP were as likely to be able to contribute as adults. However, it may have also meant that younger children missed some of the nuanced discussion because different mediums were used that may not have been accessible to them. The virtual setting also presented opportunities for greater flexibility, facilitating representation from groups who may be traditionally excluded such as those with greater health needs, poor mobility or young children [31]. However, this may have posed to a barrier to participation for those from low socioeconomic backgrounds, those with no access to technology, or those who were technology illiterate. Another strength of the online event was that participants joined from across the country, which would not have been possible in a face-to-face setting due to limited travel funding.

Participants of both the large group PPI event and the monthly PPI group series varied vastly in age, making it challenging to tailor the activities and discussions to all participants. There is a risk of over-burdening CYP with unreasonable expectations of contribution [32]. To minimise this, the initiative held multiple short sessions with frequent breaks and encouraged CYP to creatively express themselves in whichever way they felt best, or forego participation if they did not feel ready. It is the role of the research team to find innovative ways in which children with a range of abilities and levels of understanding can meaningfully contribute to the research process [2]. Ensuring diversity has been described a salient action for ensuring effective PPI [33] and the participants felt that more diversity within the genders of the CYP would have allowed the group to be more representative.

There was also diversity in previous experience with glucocorticoid medication, which informed participants’ opinions and outlooks. The researchers laid guidelines for respectful communication prior to starting discussion to create a safe and respectful space for people to contribute. The large group event began with online polls and quizzes to facilitate discussion and then split into smaller groups, in Zoom breakout rooms, for further topic exploration. The monthly PPI group began with allowing time for members to get to know each other, independent of their experience with glucocorticoid medication. The diversity of the participants ultimately became one of the strengths of the PPI group and participants reported that they found hearing each other’s experiences and forming positive relationships to be one of the most beneficial aspects of taking part. The research team also reported that they had gained new insight and in this way, PPI initiatives such as this one can help align patient and clinician perspectives.

The use of PROMs in healthcare also fosters a shift towards a patient-centred focus from a clinician/researcher centred-focus. Despite an acknowledgment that patient involvement is necessary to PROM development, few take the approach of this initiative where PPI forms an integral part of every part of the PROM development process. Wiering et al. [8] reported in a scoping review of patient involvement in PROMs that only 6.7% of studies reviewed featured patient involvement in all aspects of development.

The monthly PPI group also informed the development of a conceptual model based on their lived experience and designed key aspects of a future study developing a glucocorticoid-associated PROM. The key findings from the group have determined the scope of the PROM, identified key barriers to diverse recruitment within this patient group, and designed interviews to maximise value of input from CYP who participate. The research team held honest and open dialogue that the group’s proposals must be balanced with feasibility and scientific rigour. Here the group’s training on how research was conducted and the positive relationships between the research team and the PPI group fostered a collaborative, problem-solving approach. Ongoing input from the PPI group will continue to inform further PROM development, such as analysis of data, design of further studies (e.g. psychometric surveys or cross-cultural validation studies) and dissemination of findings [9].

## Conclusion

High quality PPI initiatives that engage with patients with lived experience of an illness or medication from the design stage of a research study, provide a valuable way for families to share their perspectives. They can be a useful educational tool to disseminate research findings that are relevant to patients and improve health research knowledge. Families found the experience to be beneficial and enjoyed the opportunity to share experiences of the effect of glucocorticoid medication on health-related quality of life. The monthly PPI group identified a need for a PROM related to glucocorticoid medication use and designed key aspects of a research study developing this.

## Supplementary Information

**Additional file 1.** Mind map of priorities in steroid research treatment generated following discussion in monthly PPI group.

**Additional file 2.** Plain Language Summary of this PPI initiative.

## Data Availability

The datasets used and/or analysed during the current study are available from the corresponding author on reasonable request.

## Abbreviations

ABPA: Allergic Bronchopulmonary Aspergillosis
CYP: Children and young people
CF: Cystic Fibrosis
HRQOL: Health-related quality of life
JIA: Juvenile Idiopathic Arthritis
PPI: Patient and public involvement
PROM: Patient reported outcome
SLE: Systemic Lupus Erythematous
