# Supplementary figures and images for: The importance of children and young person involvement in scoping the need for a paediatric glucocorticoid-associated patient reported outcome measure
Source: BMC Rheumatol. 2022 Oct 15;6:80. doi: 10.1186/s41927-022-00312-9 (PMC9568975; doi:10.1186/s41927-022-00312-9)

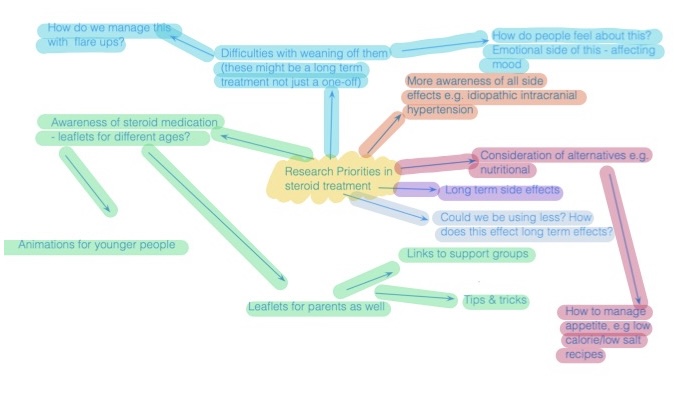

Supplement: Supplementary file 1 — Additional file 1. Mind map of priorities in steroid research treatment generated following discussion in monthly PPI group. [file 41927_2022_312_MOESM1_ESM.jpg]
