# Supplementary material for: The importance of children and young person involvement in scoping the need for a paediatric glucocorticoid-associated patient reported outcome measure
Source: BMC Rheumatol. 2022 Oct 15;6:80. doi: 10.1186/s41927-022-00312-9 (PMC9568975; doi:10.1186/s41927-022-00312-9)
Supplement: Supplementary file 2 — Additional file 2. Plain Language Summary of this PPI initiative. [file 41927_2022_312_MOESM2_ESM.docx]

Plain Language Summary

Many children and young people with rheumatological diseases have to regularly take steroids (also known as glucocorticoid medication). Steroids can work quickly and can be very effective in stopping symptoms of the disease, but they have a number of side effects that limit their long-term use. These side effects include weight gain, skin changes and mood swings. These side effects can have a negative effect on children and young people’s quality of life.

The research team hosted an online event to let children, young people and their parents know about some of from previous steroid medication related research studies. The event was organised in a way that encouraged families to share their experiences of life with steroid treatment through interactive polls and small group discussion.

Following this, a monthly group was held where children and young people could give their input into different research studies associated with steroids. They received education and training into what makes a good research study. The group co-designed a future study developing a patient-reported outcome measure about steroids. A patient-reported outcome measure is a questionnaire or series of questions that allow patients to report what matters most to them in a research or healthcare setting.

Feedback from online questionnaires showed that participants enjoyed attending the large group event and 86% would recommend it to others. They felt more confident knowing what steroids were, how they worked and what research around steroids was available. They particularly enjoyed hearing about other people’s experience and getting to share their own experience.
